# Local attributable burden disease to PM _2.5_ ambient air pollution in Medellín, Colombia, 2010–2016

**DOI:** 10.12688/f1000research.52025.2

**Published:** 2021-12-06

**Authors:** Hugo Grisales-Romero, Juan Gabriel Piñeros-Jiménez, Emmanuel Nieto, Sandra Porras-Cataño, Nora Montealegre, Difariney González, Dorian Ospina

**Affiliations:** 1 University of Antioquia, Medellin, Colombia

**Keywords:** Disability Adjusted Life Years, Population Attributable Fraction, air pollution, attributable burden, Colombian population

## Abstract

**Background:** Exposure to 2.5-micron diameter air pollutants (PM _2.5_) has been associated with an increased risk of illness and death worldwide; however, in Latin American health impacts assessment of this risk factor is scarce. Medellín is one of the most polluted cities in the region, with a population growth rate that is twice as high as that of other Colombian cities, which implies a growing population at risk.

**Methods:** A descriptive study of the disease burden was carried out using the city as the unit of observation. Health events were selected based on epidemiologic evidence and the availability of the population attributable fraction associated with PM _2.5. _The mortality records were taken from the module of deceased of the Single Registry of Affiliates of the Health System; the morbidity records were taken from the Individual Health Services Registries. For the estimation of the burden of disease, the current Global Burden of Disease guidelines were followed.

**Results:** Attributable disability-adjusted life years to exposure to ambient PM _2.5_ pollution (DALYs _PM2.5_) constituted 13.8% of total burden of the city. Males showed the greatest loss of DALYs _PM2.5_ due to acute events, while in women the greatest loss was due to chronic events. Ischemic heart disease, chronic diseases of the lower respiratory tract, and influenza and pneumonia were the events that contributed the most to DALYs _PM2.5_. 71.4% of the DALYs _PM2.5_ corresponded to mortality, mainly in the population over 65 years of age. Regarding attributable morbidity, acute events were more prevalent in both sexes, especially due to respiratory diseases

**Conclusion:** Premature death among the elderly population has the greatest weight on burden of disease attributable to ambient PM _2.5_ pollution, mainly due to respiratory and cardiovascular diseases, without significant differences according to gender.

## Introduction

Air pollution is one of the main concerns surrounding public health worldwide due to its impacts on human health and ecosystem ( Dominici *et al.*, 2006). According to the Environmental Performance Index of Yale University, poor air quality is the greatest environmental threat and the most difficult challenge for public policies in middle- and low-income countries ( Wendling *et al.*, 2020). The attributable disease burden to ambient air pollution at a global, regional, and local level has been widely documented, this involves the measurement of disability-adjusted life years (DALYs), an aggregate value of years of life lost (YLLs) due to premature death and years lived with disability (YLDs) (HEI, 2019). Globally, the Institute for Health Metrics and Evaluation (IHME) has identified to air pollution as the fifth main health risk factor for the population, and it is estimated that the exposure to PM _2.5_ contributes to 4.9 million deaths (8.7% of all deaths worldwide), and the loss of 147 million of healthy life years (5.9% of all DALYs). The main causes of mortality worldwide due to air pollution are ischemic heart disease (35.9%), stroke (21.1%), chronic obstructive pulmonary disease (COPD; 20.4%), acute respiratory infections (15.9%), and lung and respiratory tract cancer (6.9%) ( Cohen *et al.*, 2017). Additionally, a wide list of events is evidenced beside to lung and respiratory cancer, which includes neoplasms in different organs and systems ( Turner *et al.*, 2020).

The World Health Organization (WHO) considers air pollution to be the main environmental health risk factor for the population in the Americas ( Prüss-Ustün *et al.*, 2016) due to its impact in susceptible populations, such as children younger than 5 years old, pregnant women, and elderly people ( Piñeros-Jiménez *et al*., 2018). For Latin America and the Caribbean, it has been estimated that around 35,000 persons die annually to urban air pollution and 276,000 annual healthy life years are lost ( Romieu *et al*., 2012).

In Colombia, few studies on ambient air pollution epidemiology have been carried out: a review of literature between 2008 and 2016 ( Piñeros-Jiménez *et al*., 2018), 19 works were identified, which were mainly focused on the population-based analysis of the risk associated with the exposure to air pollutant on morbidity and mortality due to cardiovascular and respiratory events ( Blanco-Becerra *et al.*, 2014; Rodríguez-Villamizar *et al.*, 2010; Salazar-Ceballos & Álvarez-Miño, 2011; Gaviria *et al*., 2011). Recently Rodríguez-Villamizar *et al.* (2018) conducted a multicity ecological time-series analysis with data from four major cities in the country. This analysis found for NO _2_, PM _10_, and PM _2.5,_ statistically significant percentage increases in emergency department visits for respiratory diseases in children between 5 and 9 years old, and for circulatory diseases in persons over 60 years of age.

Regarding studies of burden of disease attributable to ambient particulate matter pollution, few robust studies have been conducted ( Romieu *et al*., 2012). In 2016, the Colombian National Health Institute estimated that 8% of 200,000 annual deaths in the country could be attributed to environmental risk factors, and calculated that 13.9% of ischemic heart disease deaths and 17.6% of chronic obstructive pulmonary disease deaths could be associated to ambient particulate matter pollution ( INS, 2018). Also, the World Bank (WB) estimated 5,000 premature deaths and 69 million DALYs annually between 2002 and 2010 ( Golub *et al*., 2014). These studies provided meaningful results of impact of air pollution in health in Colombia; however, they did not determine the magnitude of burden disease at the municipal levels where environmental phenomenon presents different magnitudes and particular local dynamics.

Medellín is the second largest city in Colombia and one the most polluted in Latin America. Since 2016, Piñeros-Jiménez *et al*., have conducted several researches about health impacts associated with short-term exposure to PM _10_, PM _2.5_ and ozone ( Piñeros-Jiménez *et al*., 2018; Piñeros-Jiménez *et al.*, 2019). They developed traceability techniques based on analytical methods to identify health events from the records of different sources of health information, which allowed them to have a more precise epidemiological baseline for the measurement of health impacts. For Medellín, an ecological time series study found that a 10 μg/m ^3^ increase in PM _2.5_ was associated with increase of 25.2% in the risk of respiratory diseases for children younger than 5 years old, and of 29.7% for adults of 65 years of age and over ( Piñeros-Jiménez *et al*., 2018). Furthermore, they could establish the increases in the risk percentage of emergency room visits for asthma (2.8%), acute respiratory infection (2.0%) and pneumonia (2.2%) due to population exposure to critical air pollution episodes occurred in February and March 2015 (Nieto-López *et al*., 2020).

Despite the advances in air pollution epidemiology research in Latin America, it has been recognized that establishing the attributable burden of disease to this environmental risk factor at the municipal level is a challenge for the academy and local management of environmental health, which requires updated information of the best quality to guide political decision-making and public planning with a territorial perspective. The aim of this study was to determine the local burden of disease to PM _2.5_ (LBD _PM2.5_) for Medellín, for which an updated epidemiological baseline focused on respiratory and circulatory events.

## Methods

### Type of study and population

A descriptive study was conducted using the city as the unit of observation. The study population were all the residents in Medellín, the second major city in Colombia, between 1 January 2010 and 31 December 2016. Medellín is located in Aburra Valley in the Andes Mountains to the central-western of Colombia and an estimated population of around 2.9 million people. Additionally, it has the fastest demographic growth in the country, with a growth rate twice that of other cities.

### Air pollutant data analysis

PM _2.5_ data were obtained from the air-quality monitoring network in Medellín. These data correspond to validated and adjusted information from eight monitoring stations distributed across the city between 1 January 2008 and 31 December 2016. Daily 24-h averages of PM _2.5_ were calculated. Data quality analysis identified gaps in each available pollutant dataset which were filled with the R package nnet (RRID:SCR_001905) for data imputation using an artificial neural network (Villa-Garzon, 2018). This package was also used for to obtain a unique assembled dataset for PM _2.5_ that represents the air pollution exposure for Medellín from information of different air monitoring stations. Daily, monthly and annually average concentrations of PM _2.5_ were calculated from this dataset.

With regard to the air pollutant data, in previous studies carried out by the authors ( Piñeros-Jiménez *et al.*, 2018), the quality analysis of the daily concentrations of PM _2.5_ available in the Intelligent Information System and the Air Quality Surveillance Network (SIATA, acronym in Spanish) of the environmental authority of the metropolitan region of Aburra Valley was made. The quality of the raw data measured in each station is carried out by the Institute of Hydrology, Meteorology and Environmental Studies (IDEAM, acronym in Spanish), who are in charge of identifying the outlier data, as well as establishing the concentration of daily averages, which are calculated with at least 75% of the hourly data valid for each day in the case of automated stations.

For each dataset, the percentage of days without available information was established and the missing daily concentrations of PM _2.5_ were determined using multiple imputation techniques by neural networks ( Villa-Garzon *et al.*, 2018). Starting from the imputed series, a representative data series of the city was assembled for each year of study, previous correlation analysis by measuring the entropy of the information based on the Bhattacharya-Hellinger-Matusita distance that allows determining the relative closeness between data sets ( Li, 2015). The results presented in this manuscript show an overview of PM _2.5_ pollution in Medellín from assembled series, with the proper quality product of a necessary purification process that ideally shows the behavior of the pollutant in the city.

### Data source and procedures for health information

All available data on morbidity and mortality population residing in Medellín during the study period were used. Data of individual deaths was obtained from death records in the deceased module of the Single Registry of Affiliates of the Health System (Registro *Único de Afiliados, módulo de Defunciones* (RUAF-D)). Information related to emergency department visits, medical outpatient services visits, and hospitalizations were obtained of individual records on the Provision of Health Services (Registros *Individuales de Prestación de Servicios de Salud* (RIPS)). All data were provided by the Social Protection System (Sistema *Integrado de la Información de la Protección Social* (SISPRO)). The records of people whose basic cause of death or main medical diagnosis was an event related to air pollution were included, following International Classification of Disease, ICD-10.

The acute events studied were ischemic heart disease (I20–I25), cerebrovascular diseases (I60–I69), influenza and pneumonia (J09–J18), and others acute lower respiratory tract infection (J20–J22). The chronic events studied were malignant neoplasms of respiratory and intrathoracic organs (C30–C39), *in situ* neoplasms (D00–D09), and chronic lower respiratory tract diseases (J40–J47).

For each health data source, a quality assessment of data was performed, considering the dimensions of completeness, consistency, accuracy, duplication and integrity for each individual record that complied with the inclusion criteria such as place of residence, year of death/medical attention, and causes of morbidity and mortality selected for this study. Underreporting of deaths was estimated with the Preston and Coale method, and the PAHO method of proportional distribution to address potential information biases (ONU, 1986).

In the mortality analysis carried out in this study, the causes that have been identified as residual or garbage codes were not considered. It was limited to the groups of causes that have been identified in the literature as being related to air pollution and for which the fraction of risk attributable to the population. To define the ICD-10 codes of the events to be included in the study, a homologation and mapping was carried out with respect to the list of causes of GBD, this activity was also validated by two clinical experts.

Recognizing the possible information biases derived from the erroneous assignment of codes of the basic causes of death by the health personnel, the process carried out allows the comparability of the results with other studies of the environmental burden of the disease.

For morbidity, underreporting could not be controlled because there was no other source of information that could contrast the source used. The reference population were people living in Medellín based on time, age, and gender criteria, according to projection of the population census published by the National Department of Statistics of Colombia.

Traceability strategies were defined for each event in order to identify prevalent cases in each year of analysis. These were previously designed by the research team for local studies based on secondary data ( Piñeros-Jiménez, 2018), which used the descriptive model of the natural history of disease, which presents the course of all biological events, the sequential action of causes (etiology), the evolution of the disease and its outcomes (recovery, chronicity, disability, or death), as well as the pre-pathogenesis and pathogenesis phases of the disease. Annual event per patient was included and point-prevalence was estimated.

### Determining burden of disease

WHO’s methodology for estimation of burden of disease was used ( WHO 2017). DALYs calculation incorporated the number of YLLs due to premature death and the number of YLDs ( Rutstein *et al*., 1983). YLLs estimation used the standard method, which includes all deaths at any age within the total estimated disease burden. As a standard value, the frontier national life expectancy projected for the year 2050 was considered, with a life expectancy at birth (LEAB) of 91.9 years for both men and women (Murray, 1995). The equation used for calculations was: $\mathrm{YLL}=\sum_{x=0}^{L}\mathrm{dx}\;e_{x}^{*}$ , where L = the ultimate age of survivors; *x* = age of death; d *x* = number of deaths at *x* age in years; e *_x_* * = life expectancy at each age based on an ideal standard.

Due to the availability of aggregated data by cause of death according to sex, age group and year, the class mark was defined as the representative value of all age intervals in the calculation of the indicator. Premature death was calculated with the difference between the class mark of the respective age group and the LEAB standard value for each one record of the database.

For calculating YLDs, the methodology of 2013 GBD guidelines by WHO was used ( WHO 2017; Salomon *et al.*, 2015), not including discounting rate of 3% and age weights. For each study event, the distribution of cases in each year were calculated according to gender and age group. Them these were divided by the number of inhabitants in Medellín in order to find the point prevalence. The disability weights per event were calculated as the following equation: $\mathrm{YLD}=D_{j}\times p_{j}\;$ where $D_{j}$ = the disability weight for each individual *j* cause, and $p_{j}$ = the prevalence of the *j* ^th^ disease. Therefore, the total of annual YLDs per event in the study period corresponds to the sum of individual YLDs per age group and gender.

DALYs were obtained from the sum of the total number of YLLs due to premature death and the total number of YLDs per year, gender, age group, and subgroup of diagnostic cause for each type of event (acute or chronic).

### Estimating local burden disease to PM _2.5_ pollution

To determine the magnitude of LBD _PM2.5_, exposure is expressed as the fraction of disease or death to the risk factor in a population and referred to as the population-attributable fraction (PAF). Due to this requirement, in the case of Medellín, it began by defining the events to study. Air pollution-associated events were limited to those causes that have been examined in GBD studies, which already had PAF data for PM _2.5_ pollution, according to the IHME measurement results for Colombia ( Cohen *et al.*, 2017).

After obtaining the frequency of YLLs, YLDs, and DALYs per event, LBD _PM2.5_ was calculated. This was done by considering the standardized PAF by age estimated for Colombia ( Cohen *et al.* 2017) in relation to each one of the diagnostic groups examined in this study. LBD _PM2.5_ was estimated by using the following equation: LBD _PM2.5_ = (YLLs or YLDs or DAILYs) × PAF _PM2.5_ ( Cohen *et al*., 2017; Grisales-Romero *et al*., 2021).

Results are shown with absolute and relative frequencies along with rates/indices of each indicator considered with a constant value of 100,000 according to Medellín’s population for each year. They are complemented, where necessary, with the 95% uncertainty interval (95% UI) generated using the Bootstrap method, a resampling technique ( Efron & Tibshirani, 1993). Data capture, storage, and processing was performed using the database management software pgAdmin 4 v2.1 ^®^ (RRID: SCR_021066). For the generation of results and graphs, the commercial software, Microsoft Excel ^®^ (RRID:SCR_016137) was used. A free office suite alternative that could also be used for this process would be LibreOffice, which is available at https://www.libreoffice.org.

### Research ethics

This project was approved by the Ethics Committee of the National School of Public Health of *Universidad de Antioquia* as declared in minutes No. 141 of April 29, 2016. All procedures performed in this project followed ethical standards contemplated in Resolution 8430 of the Ministry of Health and Social Protection of Colombia, and International Ethical Guidelines for Health-related Research Involving Humans of 2016 of the Council for International Organizations of Medical Sciences. At all times, this study used anonymized data that had the authorization of each of the sources in charge of its custody.

## Results

### Ambient PM _2.5_ pollution in Medellín

Between 2010 and 2016, daily concentrations of PM _2.5_ were 35.8 μg/m ^3^ (min–max: 13.6–123.1 μg/m ^3^). with important variations at each year, we could observe that, in 2016, there was an annual increase of the average PM _2.5_ of 21.9%, compared with the base year, 2010. Interestingly, at all years, the annually average values of PM _2.5_ were higher than the 25 μg/m ^3^ value established by the Environmental National Authority (i.e., Resolution No. 610 of 2010).

On February and March of each year the highest averages were presented during the study years, except for 2012. Likewise, there is a greater tendency to increase the monthly average for PM _2.5_ for these months during the study period. In March 2016, the highest mean value of the entire time series of data were identified.

**Figure 1.**
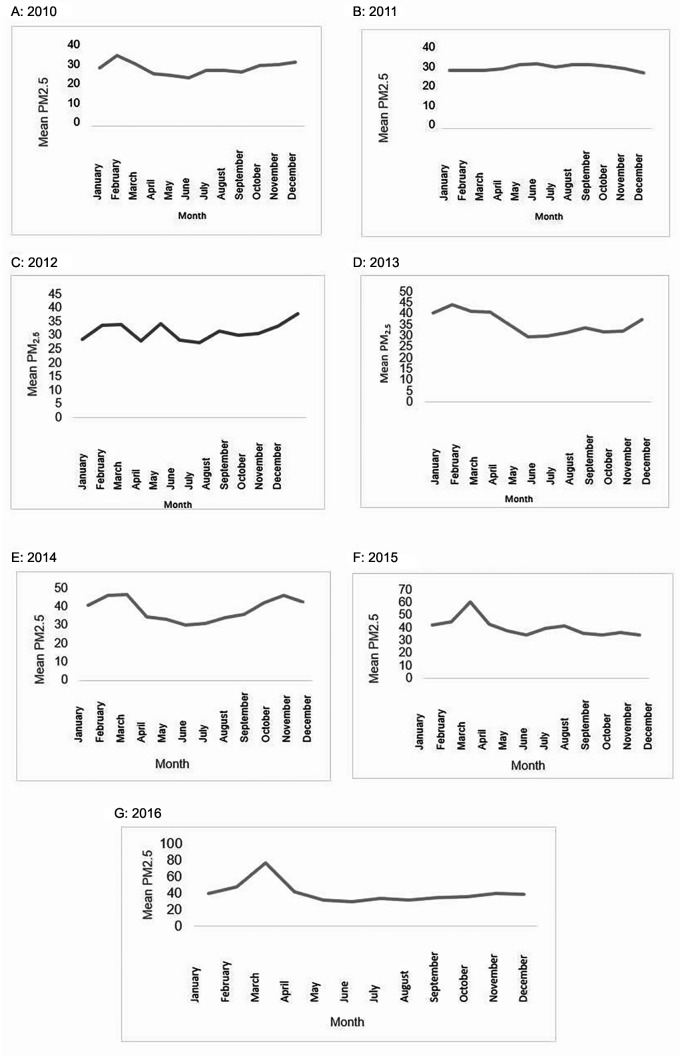
Monthly average of PM _2.5_, 2010–2016.

On the other hand, the months of October and November from 2010 to 2016 presented monthly averages with slight increases, except for 2011 ( Figure 1A–G).

### Mortality and morbidity to PM _2.5_ pollution

There was a total 28,678 of deaths for acute and chronic diseases associated with air pollution in Medellín between 2010 and 2016, of which 3,873 deaths (13.5%) were attributed to PM _2.5_ pollution. The attributable deaths to PM _2.5_ pollution per year were similar during the seven years of studied period. 61.7% (n = 2,391) of them were for acute events, of which 75% (n = 1,793) were due to circulatory system diseases, mainly ischemic heart diseases (n = 1,550). Among diseases of the respiratory system, influenza and pneumonia showed the highest frequency in the study period (n = 598). Chronic lower respiratory tract diseases were the most frequent (n = 1081) of chronic events; 95.7% corresponded to chronic obstructive pulmonary diseases ( Table 1).

**Table 1.** Attributable deaths and mortality rates to ambient PM _2.5_ pollution, 2010-2016.

Events (CIE-10 code)		2010		2011		2012		2013		2014		2015		2016	
		n (%)	MR ^**a**^	n (%)	MR	n (%)	MR	n (%)	MR	n (%)	MR	n (%)	MR	n (%)	MR
**ACUTE EVENTS**	**Circulatory system diseases**	246 (46.8)	10.5	238 (45.3)	10.1	266 (50.1)	11.1	262 (48.3)	10.8	268 (47.1)	11.0	250 (43.6)	10.2	263 (43.4)	10.6
	Ischemic heart disease (I20-I25)	210 (39.9)	9.0	209 (39.8)	8.8	231 (43.5)	9.7	227 (41.8)	9.4	231 (40.6)	9.5	212 (36.9)	8.6	230 (38.0)	9.2
	Cerebrovascular diseases (I60–I69)	36 (6.8)	1.5	29 (5.5)	1.2	35 (6.6)	1.5	35 (6.5)	1.5	37 (6.5)	1.5	38 (6.6)	1.6	33 (5.5)	1.3
	**Respiratory system diseases**	81 (15.4)	3.5	82 (15.6)	3.5	66 (12.4)	2.7	78 (14.4)	3.2	83 (14.6)	3.4	99 (17.2)	4.0	109 (18.0)	4.4
	Influenza [flu] and pneumonia (J09–J18)	81 (15.4)	3.4	81 (15.4)	3.4	64 (12.1)	2.7	77 (14.2)	3.2	82 (14.4)	3.4	97 (16.9)	3.9	108 (17.9)	4.4
	Other acute lower respiratory tract infection (J20–J22)	0	0.0	1 (0.2)	0.03	2 (0.4)	0.1	1 (0.2)	0.0	1 (0.2)	0.0	2 (0.3)	0.1	1 (0.2)	0.0
	Total	327		320		332		340		351		349		372	
**CHRONIC EVENTS**	**Respiratory system diseases**	149 (28.3)	6.4	154 (29.3)	6.5	144 (27.1)	6.0	148 (27.3)	6.1	156 (27.4)	6.4	161 (28.0)	6.5	169 (27.9)	6.8
	Chronic lower respiratory tract diseases (J40–J47)	149 (28.3)	6.4	154 (29.3)	6.5	144 (27.1)	6.0	148 (27.3)	6.1	156 (27.4)	6.4	161 (28.0)	6.5	169 (27.9)	6.8
	***Tumors [Neoplasms*]**	50 (9.5)	2.2	51 (9.7)	2.2	55 (10.4)	2.3	55 (10.1)	2.3	62 (10.9)	2.5	64 (11.1)	2.6	64 (10.6)	2.6
	Malignant neoplasms of respiratory and intrathoracic organs (C30–C39)	50 (9.5)	2.2	51 (9.7)	2.2	55 (10.4)	2.3	55 (10.1)	2.3	62 (10.9)	2.5	64 (11.1)	2.6	64 (10.6)	2.6
	Total	199		205		199		203		218		225		233	
Total		526 (13.6)	*22.5*	525 (13.6)	22.2	531 (13.7)	22.2	543 (14.0)	22.5	569 (14.7)	23.3	574 (14.8)	23.3	605 (15.6)	24.3

^a^ Mortality rate per 100,000 inhabitants.

During the study period, 567,505 prevalent cases for events associated with air pollution were identified, 75.6% (n = 428,858) were acute events, of which 92.3% (395,761) were due to respiratory system diseases. 88,083 (15.5%) prevalent cases were attributed to PM _2.5_ pollution. Respiratory system diseases were the most frequent for acute attributable events, mainly for acute lower respiratory tract infections (n = 39,163). Among chronic events, the most frequent were chronic lower respiratory tract diseases (n = 21,479) ( Table 2).

**Table 2.** Attributable prevalent cases to ambient PM _2.5_ pollution, 2010-2016.

Attributable Events		2010	2011	2012	2013	2014	2015	2016	Total
		n (%)	n (%)	n (%)	n (%)	n (%)	n (%)	n (%)	n (%)
**A** **C** **U** **T** **E** **E** **V** **E** **N** **T** **S**	**Circulatory system diseases**	455 (3.7)	434 (3.3)	594 (4.5)	626 (4.8)	585 (5.1)	574 (5.0)	544 (4.7)	3,812 (4.4)
	Ischemic heart disease (I20-I25)	245 (2.0)	235 (1.8)	283 (2.1)	297 (2.3)	264 (2.3)	259 (2.3)	251 (2.2)	1,833 (2.1)
	Cerebrovascular disease (I60–I69)	210 (1.7)	199 (1.5)	310 (2.4)	329 (2.5)	321 (2.8)	315 (2.8)	293 (2.5)	1,978 (2.3)
	**Respiratory system diseases**	8,680 (71.0)	9,287 (70.4)	9,045 (68.6)	8,657 (66.7)	8,092 (70.3)	7,982 (69.7)	8,413 (72.8)	60,156 (69.9)
	Influenza [flu] and pneumonia (J09–J18)	3,179 (26.0)	3,348 (25.4)	3,258 (24.7)	3,082 (23.8)	2,843 (24.7)	2,744 (23.9)	2,538 (22.0)	20,992 (24.4)
	Other acute lower respiratory tract infection (J20–J22)	5,500 (45.0)	5,939 (45.0)	5,787 (43.9)	5,575 (43.0)	5,249 (45.6)	5,239 (45.7)	5,875 (50.9)	39,163 (45.5)
**C** **H** **R** **O** **N** **I** **C** **E** **V** **E** **N** **T** **S**	**Respiratory system diseases**	3,007 (24.6)	3,393 (25.7)	3,439 (26.1)	3,598 (27.7)	2,727 (23.7)	2,805 (24.5)	2,510 (21.7)	21,479 (25.0)
	Chronic lower respiratory tract diseases (J40–J47)	3,007 (24.6)	3,393 (25.7)	3,439 (26.1)	3,598 (27.7)	2,727 (23.7)	2,805 (24.5)	2,510 (21.7)	21,479 (25.0)
	***Tumors [Neoplasms*]**	78 (0.6)	70 (0.5)	113 (0.9)	95 (0.7)	101 (0.9)	94 (0.8)	86 (0.7)	636 (0.7)
	Malignant neoplasms of respiratory and intrathoracic organs (C30–C39)	78 (0.6)	70 (0.5)	113 (0.9)	95 (0.7)	101 (0.9)	94 (0.8)	86 (0.7)	636 (0.7)
Total		12,219 (14.2)	13,184 (15.3)	13,191 (15.3)	12,975 (15.1)	11,504 (13.4)	11,456 (13.3)	11,554 (13.4)	86,083

### Local attributable burden of disease

**Figure 2.**
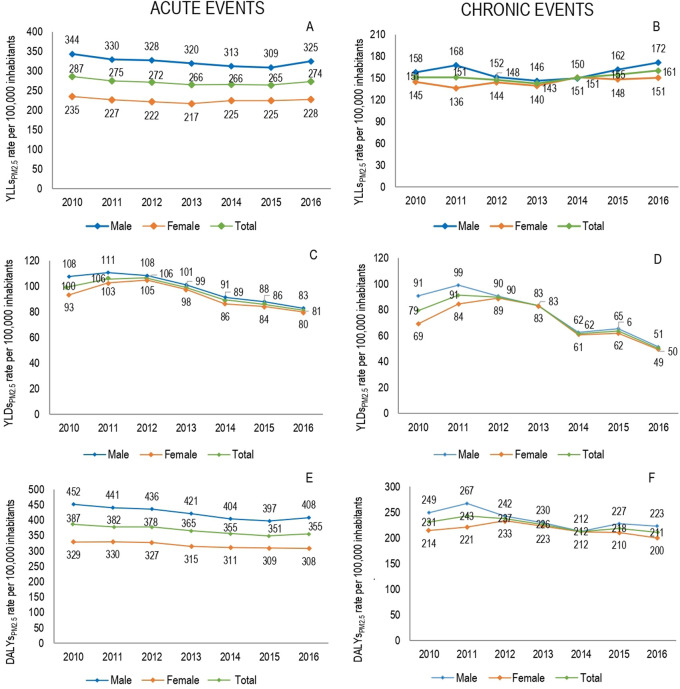
Attributable local burden disease to ambient PM _2.5_ pollution by gender, 2010-2016. YLLs _PM2.5:_ Attributable years of life lost to ambient PM _2.5_ pollution. YLDs _PM2.5:_ Attributable years lived with disability to ambient PM _2.5_ pollution. DALYs _PM2.5_: Attributable disability adjusted life years to ambient PM _2.5_ pollution.

During the seven-year period there was a premature YLLs of 536,772 (95% UI 524,048–549,495) due to events related to air pollution in Medellín; 13.0% (n = 71,590 (95% UI 69,843–73,339)) corresponded to LBD _PM2.5_ ( Table 3). The attributable YLLs to PM _2.5_ pollution (YLLs _PM2.5_) showed little variation throughout the period, with an annual average of attributable mortality burden calculated in 10,227 (95% UI 9,950–10,505) of YLLs. The Figure 2A–B shows annual YLLs _PM2.5_ rates for males and females between 2010 and 2016. In both genders, stable losses were observed in the YLLPs _PM2.5_ rates with a tendency to decrease. The highest concentration of YLLs _PM2.5_ were presented in 2016, with annual rates for this year of 496.9 per 100,000 inhabitants for males and 378.6 cases per 100,000 inhabitants for females.

**Table 3.** Attributable local burden disease to ambient PM _2.5_ pollution by type of event and gender, 2010-2016.

Type of event	Gender	LBD ^***a***^	LBD _**PM2.5**_ ^***b***^	% LBD _**PM2.5**_	Average LBD _**PM2.5**_ rate	Average LDB _**PM2.5**_ (UI 95%) ^***c***^
**A** **C** **U** **T** **E** **E** **V** **E** **N** **T** **S**	Male					
	YLLs	197,533	25,795	13.1	323.8	3,685 (3,609 – 3,761)
	YLDs	55,016	7,838	14.2	688.8	1,120 (1,030 – 1,209)
	DALYs	252,550	33,633	13.3	422.2	4,806 (4,684 – 4,925)
	Female					
	YLLs	156,458	20,201	12.9	225.6	2,886 (2,820 – 2,952)
	YLDs	57,124	8,286	14.5	647.6	1,183 (1,095 – 1,273)
	DALYs	213,582	28,487	13.3	318.1	4,070 (4,027 – 4,113)
	Total					
	YLLs	353,991	45,996	13.0	271.8	6,570 (6,454 – 6,688)
	YLDs	112,140	16,124	14.4	422.2	2,302 (2,129 – 2,477)
	DALYs	466,132	62,120	13.3	367.1	8,873 (8,716 – 9,032)
**C** **H** **R** **O** **N** **I** **C** **E** **V** **E** **N** **T** **S**	Male					
	YLLs	91,173	12,600	13.8	158.2	1,799 (1,694 – 1,906)
	YLDs	38,125	6,147	16.1	540.1	879 (719 – 1,038)
	DALYs	129,298	18,747	14.5	235.3	2,679 (2,537 – 2,819)
	Female					
	YLLs	91,608	12,994	14.2	145.1	1,857 (1,768 – 1,945)
	YLDs	39,315	6,347	16.1	496.1	906 (753 – 1,060)
	DALYs	130,923	19,341	14.8	216.0	2,763 (2,669 – 2,857)
	Total					
	YLLs	182,781	25,594	14.0	151.3	3,656 (3,496 – 3,817)
	YLDs	77,440	12,494	16.1	516.9	1,783 (1,484 – 2,085)
	DALYs	260,221	38,089	14.6	225.1	5,441 (5,262 – 5,621)

^a^ LBD: Local Burden Disease.

^b^ LBD _PM2.5_: Attributable Local Burden Disease to ambient PM _2.5_ pollution.

^c^ UI 95%: Uncertainty Interval of 95% In relation to the attributable total per each event.

The highest attributable mortality burden was found for acute events with 45,996 (95% UI 45,175-46,817) YLLs _PM2.5_, on the other hand, chronic events presented 25,594 (95% UI 24,454-26,734) YLLs _PM2.5_ for chronic events. Males had a greater contribution to the attributable mortality burden for acute events (56.1%), while females showed a slightly higher frequency of this for chronic events (50.8%). No large fluctuations were observed in the distribution of the attributable burden during period; although, in 2016 there was a slightly higher concentration of mortality due to the two types of events for both genders.

When viewed in term of disability, a loss of 189,580 (95% UI 168,455–210,703) YLDs due to respiratory and circulatory events related to air pollution was observed. 15.1% (n = 28,618 (95% UI 25,280–31,956)) corresponded to LBD _PM2.5_, with an annual average of 4,086 (95% UI 3,619–4,565) YLDs. The highest proportion of attributable disability loss to PM2.5 pollution (YLDs _PM2.5_) occurred in 2011 and 2012, when 32,6% of YLDs _PM2.5_ were concentrated. Compared with 2010, in 2016 the rate of YLDs _PM2.5_ decreased by 36.4%, with a more marked decrease in males ( Figure 2C–D). Acute events constituted 56.3% of YLDs _PM2.5_ (n = 16,124 (95% UI 14,902–17,346)). Females had a higher percentage contribution, both for YLDs _PM2.5_ due to acute events (51.4%) and YLDs _PM2.5_ due to chronic events (50.8%). However, throughout the study period the highest rates occurred in men.

Of total LBDs associated with pollution (n = 726,353 DALYs, 95% UI 715,045-737,659), 73.9% corresponded to YLLs and the remaining to YLDs. The premature mortality due to acute events was higher than that estimated for chronic events, with 33 percentage points of difference, with a slightly greater magnitude in the male burden than the female burden ( Table 3). Of total estimated DALYs, 13.8% (95% UI 13.7%–13.9%) was attributed to PM _2.5_ pollution (DALYs _PM2.5_), with an average of 14,315 DALYs _PM2.5_ per year (95% UI 14,005–14,626), equivalent to rate of 592.2 DALYs _PM2.5_ per 100,000 cases. Compared with the initial year of study in 2016, a decrease of 8.3% in total DALYs _PM2.5_ was observed, with decreases of 8.1%in DALYs _PM2.5_ due to acute events and 8.6% due to chronic events. According to the gender, distribution of DALYs _PM2.5_ had variations by type of event; while DALYs _PM2.5_ due to acute events was higher in males (54.1%), the DALYs due to chronic events were slightly concentrated in females (50.8%) ( Table 3) ( Figure 2E–F).

### Attributable burden to PM _2.5_ pollution by age group and type of event

LBD _PM2.5_ showed a positive gradient from 35 years of age, with significant differences among the five-year age groups. Those over 65 years of age contributed with 61.7% of the total burden, with the highest rates in the group of 80 years and older. In all the five-year age groups, LBD _PM2.5_ for acute events was higher than for chronic events, with considerable differences that were more marked until the age of 59 years. After 60 years of age, the differences were smaller although maintaining the trend. LBD _PM2.5_ for chronic events was comparable in the age groups between 15 years and 44 years; but after 45 years of age, it gradually increased until the age of 79 years, when it slowly decreases in the last age group. Only 10.3% of the total attributable burden was in the population younger than 39 years old ( Table 4). Male showed the highest prevalence of LBD _PM2.5_, with the highest difference in the 40–44 and 55–59 age groups, and an approximate ratio of 2 to 1.

**Table 4.** Attributable local burden disease to ambient PM _2.5_ pollution by age group, 2010-2016.

Age group	Chronic Events			Acute Events			Total		
	DALYs _**PM2.5**_	% DALYs _**PM2.5**_ ^***a***^	DALYs _**PM2.5**_ Rate	DALYs _**PM2.5**_	% DALYs _**PM2.5**_	DALYs _**PM2.5**_ Rate	DALYs _**PM2.5**_	% DALYs _**PM2.5**_ ^***b***^	DALYs _**PM2.5**_ Rate
0-4	123	3.2	12.0	3769	96.8	368.3	3892	3.9	380.3
5-9	26	2.6	2.5	973	97.4	92.9	999	1.0	95.4
10-14	19	4.1	1.7	442	95.9	39.5	461	0.5	41.2
15-19	105	13.5	8.3	670	86.5	53.1	775	0.8	61.5
20-24	72	11.1	5.1	579	88.9	41.2	651	0.6	46.3
25-29	110	10.1	7.8	980	89.9	69.5	1090	1.1	77.3
30-34	131	13.1	10.5	867	86.9	69.5	998	1.0	80.0
35-39	234	15.9	21.6	1,236	84.1	114.0	1,470	1.5	135.6
40-44	299	15.9	26.5	1,580	84.1	139.7	1,879	1.9	166.2
45-49	702	20.7	51.5	2,690	79.3	197.4	3,392	3.4	248.9
50-54	1,541	28.9	116.9	3,789	71.1	287.6	5,330	5.3	404.5
55-59	2,773	35.2	258.2	5,095	64.8	474.5	7,868	7.9	732.7
60-64	3,983	41.5	481.9	5,625	58.5	680.6	9,607	9.6	1,162.5
65-69	4,785	45.5	804.5	5,720	54.5	961.8	10,505	10.5	1,766.4
70-74	5,521	47.7	1,395.3	6,065	52.3	1,532.8	11,586	11.6	2,928.0
75-79	5,962	48.8	1,932.0	6,248	51.2	2,024.6	12,211	12.2	3,956.6
80 and older	11,704	42.6	3,671.5	15,790	57.4	4,953.3	27,495	27.4	8,624.8

DALYs _PM2.5_: attributable disability adjusted life years to ambient PM _2.5_ pollution.

^a^ % in relation to the total per age group.

^b^ % in relation to the total.

Five groups provided the highest contribution to attributable premature mortality and disability according to the type of event by diagnosis group (chapter) of the seven groups analyzed ( Table 5). 80.6% of premature deaths were caused by ischemic heart disease (40.1% of YLL _PM2.5_), chronic lower respiratory tract diseases (23.4% of YLL _PM2.5_) and acute infections of the lower respiratory tract (16.9% of YLL _PM2.5_). On the other hand, among the causes that most contributed to attributable disability were chronic lower respiratory tract diseases (43.3% of YLD _PM2.5_), other acute infections of the lower respiratory tract (24.3%) and influenza and pneumonia (20.1% of YLD _PM2.5_). In contrast, the group of neoplasms had a relatively lower contribution to LBD _PM2.5_, explaining 8.8% of DALYs _PM2.5_ (12.2% of YLL _PM2.5_ and 0.3% of YLD _PM2.5_).

**Table 5.** Disability adjusted life years and disease burden attributable to ambient PM _2.5_ pollution according to each group and sub-group of events, Medellín, 2010-2016.

Group of Event (CIE-10 codes)	YLLs _**PM2.5**_	YLLs _**PM2.5**_ Rate	% LBD _**PM2.5**_ ^***a***^	YLDs _**PM2.5**_	YLDs _**PM2.5**_ Rate	% LBD _**PM2.5**_ ^***a***^	DALYs _**PM2.5**_	DALYs _**PM2.5**_ Rate	± DALYs _**PM2.5**_ (95% UI) ^**b**^
ACUTE EVENTS									
Respiratory system diseases									
Influenza and pneumonia (J09–J18)	12,101	71.5	67.8	5,738	237.4	32.2	17,839	105.4	2,548 (1,148 – 3,948)
Other acute lower respiratory tract infection (J20–J22)	395	2.3	5.4	6,942	287.2	94.6	7,336	43.4	1,948 (594 – 2,594)
Circulatory system diseases									
Ischemic heart diseases (I20-I25)	28,717	169.7	93.4	2,031	84.0	6.6	30,748	181.7	4,393 (1,771 – 7,014)
Cerebrovascular diseases (I60–I69)	4,784	28.3	77.2	1,413	58.4	22.8	6,197	36.6	885 (369 – 1,401)
CHRONIC EVENTS									
Respiratory system diseases									
Chronic lower respiratory tract diseases (J40–J47)	16,852	99.6	57.6	12,400	512.9	42.4	29,252	172.9	4,179 (756 – 7.602)
Neoplasms									
*in situ* neoplasms *(D00–D09)*	0	0.0	0.0	0.2	0.0	100.0	0.2	0.0	0.032 (0.009 – 0.06)
Malignant neoplasms of respiratory and intrathoracic organs (C30–C39)	8,742	51.7	98.9	94	3.9	1.1	8,836	52.2	1,262 (552 – 1,972)

YLLs _PM2.5:_ Attributable years of life lost to ambient PM _2.5_ pollution. YLDs _PM2.5:_ Attributable years lived with disability to ambient PM _2.5_ pollution. DALYs _PM2.5_: Attributable disability adjusted life years to ambient PM _2.5_ pollution. LBD _PM2.5_: Attributable local burden disease to ambient PM _2.5_ pollution.

^a^ % in relation to the total LBD _PM2.5_ by group of event.

^b^ Period 2010-2016, Uncertainty ranges of 95% calculated using the bootstrap method for n = 10,000 samples.

**Figure 3.**
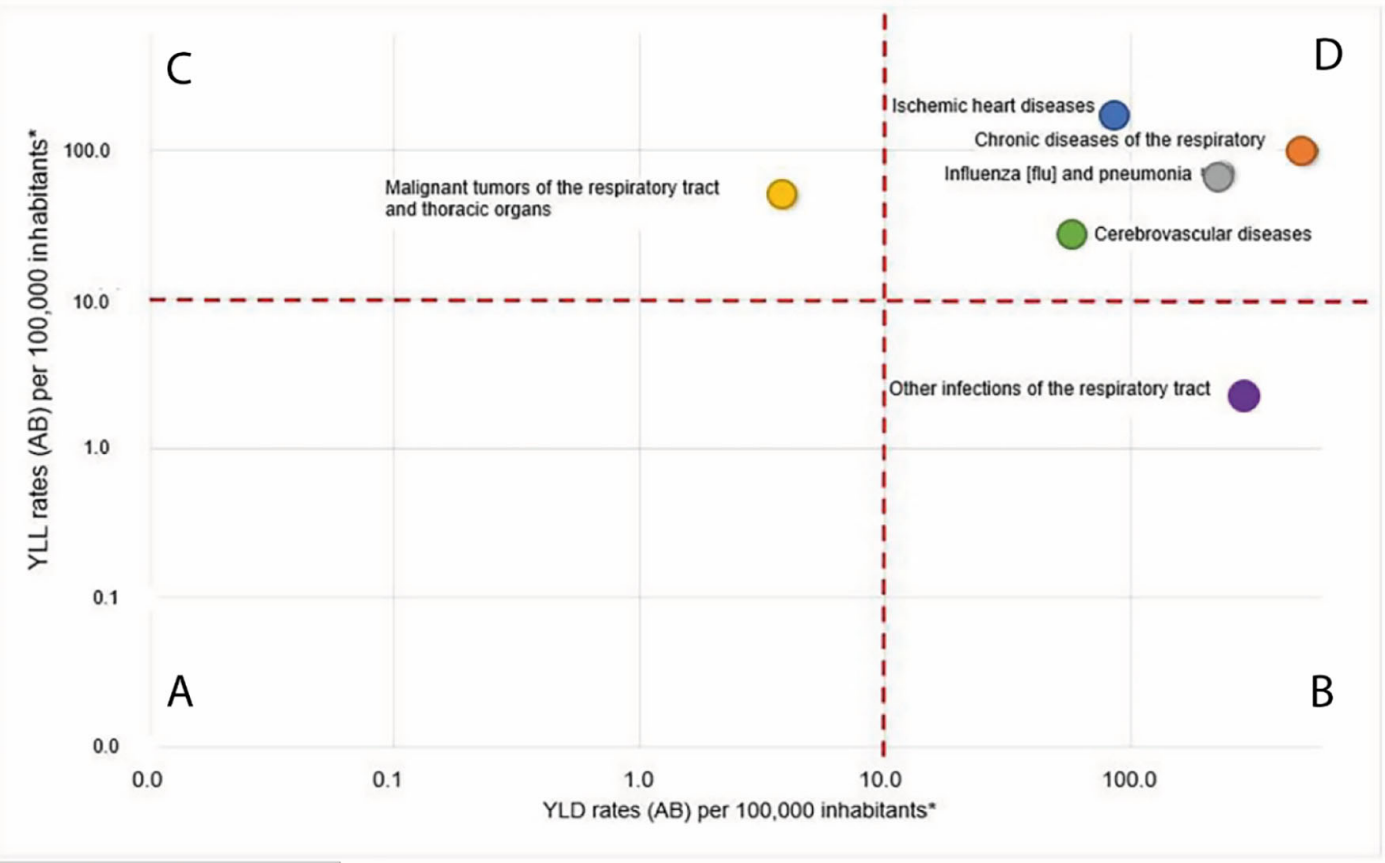
Relation of YLL _PM2.5_ and YLD _PM2.5_ rates per group of events, 2010 *–*2016. YLLs _PM2.5:_ Attributable years of life lost to ambient PM _2.5_ pollution. YLDs _PM2.5:_ Attributable years lived with disability to ambient PM _2.5_ pollution. ^a^ Logarithmic scale.

Figure 3 shows the relationship of the rates of YLL _PM2.5_ and YLD _PM2.5_ for each diagnosis group studied. The groups of causes were classified into four categories according to the relationship between the two rates: A (low mortality and low disability), B (low mortality and high disability), C (high mortality and low disability) and D (high mortality and high disability). Four of the seven groups of events studied (ischemic heart disease, cerebrovascular disease, influenza and pneumonia, and chronic lower respiratory tract diseases) were included in category D, due to their contribution to both mortality and disability. On the other hand, *in situ* neoplasms were included in category A, other acute lower respiratory tract infections in category B, and malignant neoplasms of respiratory and intrathoracic organs in category C.

Table 6 shows the ranking of the diagnostic events according to their contribution to the LBD _PM2.5_. Comparing the years of beginning and end of the study period, there were no changes in the order of the groups of events. In both years, the three highest DALY _PM2.5_ rates were found to be due to ischemic heart disease, chronic lower respiratory tract disease, and influenza and pneumonia. Cerebrovascular diseases, chronic lower respiratory tract diseases and *in situ* neoplasms showed a reduction of more than 10% in the rates of DALY _PM2.5_ by 2016 in relation to the year of initiation of the study. The opposite occurred with malignant neoplasms of respiratory and intrathoracic organs, which showed an increase of 22.3% in DALY _PM2.5_ rate.

**Table 6.** Leading of diagnosis group by attributable DALYs ambient PM _2.5_ pollution in Medellín, 2010 and 2016.

2010				Diagnosis Group	Change	2016			
Local DALYs	DALYs _**PM2.5**_	DALYs _**PM2.5**_ Rate ^***a***^	Attribution percentage ^***b***^			Local DALYs	DALYs _**PM2.5**_	DALYs _**PM2.5**_ Rate ^**a**^	Attribution percentage ^***b***^
33,561	4,420	188.6	30.6	Ischemic heart diseases	**-8.6**	32,584	4,288	172.4	30.5
25,924	4,287	183.0	29.6	Chronic lower respiratory tract diseases	**-16.7**	22,844	3,792	152.5	27.0
17,447	2,652	113.2	18.3	Influenza and pneumonia	**-4.1**	17,753	2,698	108.5	19.2
10,527	1,116	47.6	7.7	Malignant neoplasms of respiratory and intrathoracic organs	**+22.3**	13,660	1,448	58.2	10.3
6,808	1,035	44.2	7.2	Other acute lower respiratory tract infections	**-8.9**	6,579	1,000	40.2	7.1
10,314	951	40.6	6.6	Cerebrovascular diseases	**-16.4**	9,129	844	33.9	6.0
0.2	0.02	0.001	0.0	*In situ* neoplasms	**-75.1**	0.1	0.01	0.0002	0.0

DALY: Disability adjusted life years. DALYs _PM2.5_: Attributable disability adjusted life years to ambient PM _2.5_ pollution.

^a^ per 100,000 inhabitants.

^b^ % in relation to the total attributable local burden.

## Discussion

Nowadays, ambient air pollution by the criteria pollutant PM _2.5_ is considered one of the biggest environmental problems at the global and local level due to the impact it causes on the health of populations ( Apte *et al.*, 2018). Although cities in Latin America and the Caribbean region have shown annual average PM _2.5_ values that could be considered moderate if compared to cities located in Southeastern Asia and India, most of these have reported annual average higher than the levels recommended by WHO of 10 μg/m ^3^ ( Riojas, 2016). For Medellín, one of the most polluted cities of the region, the annual average PM _2.5_ levels during the seven years of study was 35.6 μg/m ^3^, where more than 90% of the days had daily averages above 25 μg/m ^3^ ( Piñeros-Jiménez *et al.*, 2018), risk factor to which approximately 3 million people are exposed. Under these conditions, in the last five years, some studies have been carried out to establish the health impacts of criteria pollutants (PM _10_, PM _2.5_, ozone and nitrogenous) with population models of a single and multiple pollutants at a local level ( Piñeros-Jiménez *et al*. 2018, Piñeros-Jiménez *et al*. 2019, Rodríguez-Villamizar *et al*. 2018). This is the first study that seeks to establish the magnitude of such an impact by using a holistic indicator.

Since the 1990s, multiple strategies have been used to gather knowledge about the burden disease caused by different risk factors. These strategies offer a holistic view of the joint effects of morbidity and mortality in the number of healthy life years lost. Environmental risk factors, mainly air pollutants, have had a growing interest among decision makers and the community at the local and global level, and have been prioritized in the political agendas promoted by research in environmental epidemiology in recent decades. Among the advances in research, the studies that analyze the magnitude of the impacts based on holistic indicators at the global and national level, supported by exposure-response functions and relative risk analysis, with the data available in the health and environmental information systems, stand out. All of which may help in transcending towards causality ( Burnett *et al.*, 2014).

Air pollution epidemiological research in Latin America and Colombia has been characterized by ecological studies of time series and some panel studies, which have confirmed the short-term effects associated to criteria pollutants ( Romieu *et al*., 2012; Piñeros-Jiménez *et al*., 2018). Very few studies have been carried out at the local level to document the local burden of disease attributable to air pollution ( Golub *et al.*, 2014; INS, 2018). No one has evaluated the long-term effects.

In this study, we provided a detailed view of the local burden of disease attributable to air pollution, for Medellín, one of most development cities of the country and region. Which recognizes the local character of the epidemiological phenomenon associated to air pollution, and the need for updated information to influence public environmental policy in the city. It was focused on using a synthetic indicator: DALYs, an aggregate value YLLs due to premature death and YLDs, based on up-to-date methodologies validated by experts and international organizations ( Cohen *et al.*, 2017; World Bank, 2016; WHO 2016). In the absence of local cohort studies, the PAF estimates made in the GBD study were used, which assumed a non-linear relationship between the incidence of health events and short- and long-term exposure to particulate matter and developed the integrated exposure–response curve to estimate the long-term PM _2.5_ exposure–response association from low exposure level to concentration as high as 1000 μg/m ^3^ to avoid overestimating the magnitude of health effects ( Burnett *et al.*, 2014)

We found that the attributable burden disease PM _2.5_ pollution constituted 13.8% of the total local burden of DALYs for all the selected pollution-related events (100,208 DALYs _PM2.5_ out of 726,352 total DALYs,). In the study on environmental burden disease in Colombia carried out by National Health Institute of Colombia (INS), for 2016, 19% of the total national burden disease was associated to environmental risk factors (air, water, and other similar factors) ( INS, 2018). Regarding air pollution, YLLs _PM2.5_ was calculated in 619.8 per 100,000 inhabitants. In our study, for the same year, 434.3 YLLs _PM2.5_ per 100,000 inhabitants was estimated. This difference can be considered reasonable, since the study cited above was carried out in a higher geographical area (national level), and it is possible that cities with a greater air pollution problem such as the capital of the country (Bogota D.C.) had a greater weight in the total estimate.

Acute diseases contributed with 62.0% of DALY _PM2.5_, this is explained by the higher contribution of YLLs _PM2.5_ (74.0%) in the burden health index, mainly by ischemic heart disease, that constitute 30.5% of DALY _PM2.5_ for 2016 in contrast to the estimation of 15.8% from the study on the environmental burden disease in Colombia for this year ( INS, 2018). Our results are consistent with the results of the study by Cohen *et al.* (2017) regarding the events but not the magnitudes. The number of DALYs associated to ischemic heart disease was the highest in this study, similar results was found in India State-Level Disease Burden Initiative Air Pollution Collaborators study for 2017 (2019). There is more and more evidence from prospective studies that PM _2.5_ exerts adverse effects particularly on the cardiovascular system, contributing substantially (mainly through mechanisms of atherosclerosis, thrombosis and inflammation) to coronary artery disease, but also to heart failure, hypertension, diabetes and cardiac arrhythmias ( Hu, *et al.*, 2018; Hajat, *et al.*, 2019; Franklin, *et al*., 2015; Kauffman, *et al.*, 2016a; Kauffman, *et al*., 2016b; Song, *et al*., 2020), which can help to explain the findings regarding this type of event in relation to the attributable burden disease.

In the case of the burden of disease attributable to acute respiratory diseases, our result differs from other studies conducted with national data. This can be observed in the estimated proportion of DALY _PM2.5_ for acute lower respiratory tract disease in the INS study was of 13.7% ( INS, 2018), which contrasts with the 7.1% reported by us for the events of the diagnostic group of other acute lower respiratory tract infection events (J20–J22) in Medellín. Regarding the study by Golub *et al.* (2014), we found more approximate results, although the basis for calculating the attributable DALYs in the case of acute respiratory disease was the proxy for the reports of respiratory symptoms. These differences could be explained by the methodology used to identify the cases and calculate the indicators. In our study we used a data mining-based traceability strategy to select health events that was constructed based on the natural history of the disease. Which established that the duration of acute respiratory events was 15 days, and with this criterion grouped the records of each source of information into a data set that made up each case ( Piñeros-Jiménez *et al*., 2018). This strategy allowed a more precise approach to the number of events and the calculation of the study indicators. This strategy allowed a more precise approach to the number of events and the calculation of the study indicators.

Chronic diseases of the lower respiratory tract constituted the chronic events with the greatest weight in the total of DALYs _PM2.5_ (38%) in our study. This result is consistent with estimations of DALYs due to chronic respiratory diseases in relation to the IHME reports. Between 2010 and 2016, the average rate of DALYs due to chronic diseases of the respiratory tract and ischemic heart diseases in Medellín, was of 172.9 and 181.7 per 100,000 inhabitants, respectively. Estimations published by the IHME show that, in 2016, around 112.2 DALYs due to chronic respiratory diseases were calculated per 100,000 inhabitants in Colombia. Also, in this period, 165.32 (106.5–217.9) DALYs due to ischemic heart disease were calculated per 100,000 inhabitants, which were associated to air pollution ( Cohen et al., 2017).

Among the events included in the diagnosis group of chronic lower respiratory tract diseases were asthma (J45) and COPD (J40–J42), two of the most relevant events in relation to the attributable burden disease to exposure to air pollution, specifically for the pollutants PM _2.5_, NO _2_ and ozone ( Achakulwisut *et al*., 2019; Anenberg *et al*., 2018, Cohen *et al*., 2017). In the case of COPD, although its impact on GBD has been established with a global point prevalence of 3.918% (95% UI (3.5111–4.3201) and a mortality rate of 41.9 deaths per 100,000 people (5.7% of all deaths from all causes) (GBD Chronic Respiratory Disease Collaborators, 2020), its risk association with some air pollutants has had a positive sign but of low magnitude and in some cases without statistical significance ( Schikowski *et al*., 2005, 2014), including its specific association with ambient PM _2.5_ pollution ( Atkinson RW *et al*., 2015; Dany Doiron *et al*., 2019). Globally, seven million deaths were attributed to the joint effects of environmental and domestic air pollution ( Cohen *et al*., 2017). And it is recognized that the effects can be divided into short and long-term effects, ranging from exacerbation of existing symptoms, impaired lung function and increase in hospitalization and mortality rates. Prolonged exposure to air with a high concentration of pollutants can also increase the incidence of COPD.

Air pollution can induce the development of asthma, increasing respiratory morbidity and mortality, particularly in minority groups ( Nishimura *et al*., 2013). Annually, it is estimated that 40 million (95% UI 18–52) of new cases of pediatric asthma could be attributable to NO _2_ contamination, with a higher burden of new asthma cases associated with NO _2_ exposure per 100,000 children for the Latin American region (340 cases per year, 95% UI 150–440) ( Achakulwisut *et al.*, 2019). Likewise, it is estimated that 9–23 million and 5–10 million annual asthma emergency room visits globally could be attributable to ozone and PM _2.5_, respectively ( Anenberg *et al.*, 2018). Our results demonstrate an important impact of ambient PM _2.5_ pollution on chronic respiratory diseases that should be analyzed in more detail, with the aim of generating useful knowledge for the design of interventions and decision-making in specific groups, especially taking into account, age groups where asthma and COPD are more frequent.

In contrast with other reports, the contribution of lung and airway cancer to local DALYs _PM2.5_ was relatively low (8.8%), and it was explained almost exclusively by YLLs _PM2.5._ The Cohen study found that lung and airway cancer accounted for 16.5% of the global burden attributable to PM _2.5_, while recently Yin *et al.* reported for China that 16.6% of deaths attributed to ambient particulate matters at the national level were due to this this cause ( Cohen *et al.*, 2017; Yin *et al.*, 2020). This difference of more than double the DALYs _PM2.5_ could be due to the level of analysis carried out in the different studies, but it is mainly explained by the coverage of high-cost disease care in our country, where there is a health system with great inequalities in access, which makes these types of diseases often go undiagnosed and unattended, so they are not reported to health information systems. This situation is especially worrying in the economically most vulnerable sectors of the population, there is a hidden burden of the disease that makes difficult to carry out targeted interventions according to equity criteria.

The highest proportion of DALYs _PM2_ was related to YLL _PM2.5_, at 71.4%. This apparent paradoxical effect of a significant premature mortality burden attributable to PM _2.5_ pollution could be explained using the protracted polarized model, which is predominant in Latin America and other regions of the world sharing the same development features. Here we find a superposition of transmissible acute diseases and non-transmissible chronic diseases, along with the reappearance of emergent diseases, which affect vulnerable human groups discriminately and disparately, as observed in the population of Medellín. Despite the improvements in the control of childhood diseases, as well as demographic changes and the increased life expectancy, conditions of inequality, poverty and extreme poverty persist, which add to the new risk factors, such as PM _2.5_ pollution caused by transportation and urbanization ( GBD 2019 Risk Factors Collaborators, 2020). This new risk factor generates a negative impact on air quality, affecting the exposed population, accelerating disease and increasing mortality, and resulting in an unbalanced pattern of death and disability.

It is worth mentioning that the effects of air pollution on health are not directly proportional to the intensity of exposure in individuals, but it is clear that such effects exist and are dependent on other variables as well as the time and level of exposure. Air quality in high-income countries has improved in recent decades, however, adverse effects of external air pollution on health due to particulate matter continue to be a public health problem worldwide, even if levels are low ( Cohen et al., 2017; Liu et al., 2021).

We can conclude that 71.4% of DALYs _PM2.5_ between 2010 and 2016 was due to premature death; especially YLLs _PM2.5_ due to acute events. A proportion of 28.6% was YLDs _PM2.5_. The greatest concentration of YLLs _PM2.5_ was associated to ischemic heart diseases and chronic lower respiratory tract diseases with a high proportion of COPD, particularly in male older than 80 years of age. Considering YLDs _PM2.5_, these were caused mainly by chronic lower respiratory tract diseases, influenza and pneumonia, and other acute lower respiratory tract infection which were more prevalent among the population older than 60 years of age, in both genders.

One of the greatest strengths of this study is that it was pioneers in Colombia and region in the use of the new proposed methodology to calculate the burden of disease indicators, as it uses the new GBD study ( GBD 2019 Risk Factors Collaborators, 2020), which gives a higher importance to prevalence over incidence as epidemiological indicators for disability calculations, this allows establishing the basis for future studies based on our findings. The quality assessment of data from the information sources we used exhaustive and included protocols that have been previously validated (Piñeros-Jiménez *et al.*, 2018; Piñeros-Jiménez *et al.*, 2019), in contrast with the majority of GBD studies where it has been shown to be one of the greatest obstacles in the application of the disease burden methodology. We obtained consistency percentages of more than 90% in morbidity aspects and more than 82% in mortality aspects, which are comparable to the results of INS and Ribotta studies ( INS, 2018; Ribotta *et al.*, 2019).

Since the first study of the global burden of disease in 1993, constant updates have been made. In 2010 they introduced significant changes in the methodology for the measurement of the base metric of the Years of Life Adjusted for Disability, which were supported by the experience of around 40 working groups in the area of epidemiology, several of them providing support to the public health programs of the WHO. Thus, over more than two decades this methodology has been refined and validated in national and international health analysis scenarios.

In this study, the latest theoretical and methodological criteria defined by the WHO since 2013 were taken into account, omitting economic and social evaluations in the metric. In addition, to identify the disability weights associated with each of the events related to air pollution, the lists of the study of the global burden of diseases 1990-2017 updated by the IHME were used and the Attributable Fractions were considered. Population-based (FAP) associated with PM _2.5_ contamination standardized by age that were estimated for Colombia in a study carried out by the IHME ( Cohen *et al.*, 2017).

Among the limitations, we can mention that the multiple sources we used respond in an unarticulated way to events follow-up, which does not allow for an optimal traceability. The effects of migration could not be considered, and it was impossible to make an adjustment of results according to the coverage of morbidity information. The underreporting of the RIPS corresponds to a reality inherent to the reporting dynamics of morbidity of Colombia’s information system, as this is recognized in some previous publications ( Castañeda-Orjuela *et al.*, 2015; Rosselli & Rueda, 2012). However, the advances in the timeliness of information that have been achieved during the last decade due to the efforts made by the health system at the local level, particularly Medellín has achieved less underreporting than other cities in the country. It should be noted that the estimation of underreporting considers the clinical history as a source of comparison for the RIPS, which as the primary document would allow comparison with the diagnoses of interest of the RIPS (capture-recapture method). However, this procedure requires access to data that is under the habeas data considerations.

We do not consider the National Demographic and Health Survey as a source of comparison with the RIPS, due to the temporal differences between the RIPS used in the present study (2010-2016) and the information from the survey, whose most current version is that of the year 2015. Added to this is the difficulty in accessing to its anonymized database.

Also, estimations of the Population Attributable Fraction proposed by the GBD methodology, which were used for a list of specific events, could not be representative for Medellín’s population. The prevalence values that we used in this study were related only with those people who visited medical services; therefore, stating that this information can be extrapolated to obtain the actual prevalence of the diseases we examined may be risky.

We recommend developing cohort studies for the local context, which can help in the documentation of the actual attribution (attributable risk estimation) of morbidity and mortality to air pollution due to the environmental risk factors that have been explained by literature. Morbidity studies should be conducted with a focus on disability analysis at a local level, to improve the estimation of the disease burden attributable to environmental pollution, based on data that accurately show those events associated to environmental factors.

## Data Availability

Zenodo: Underlying data for ‘Local attributable burden disease to PM2.5 ambient air pollution in Medellín, Colombia, 2010–2016’ https://doi.org/10.5281/zenodo.4646592 ( Grisales-Romero *et al.*, 2021). This project contains the following underlying data:
Dataset 1_YLL_YLD_DALY_Dictionary.xlsx Data are available under the terms of the Creative Commons Attribution 4.0 International license (CC-BY 4.0). The environmental data needed to replicate these analyses are publicly available at https://siata.gov.co/descarga_siata/index.php/Index2/ The health data needed to replicate these analyses are publicly available at https://www.sispro.gov.co/Pages/Home.aspx The environmental health data needed to replicate these analyses are publicly available at https://datosabiertos.metropol.gov.co/search/field_topic/medio-ambiente-3

## References

[ref1] Achakulwisut P Brauer M Hystad P : Global, national, and urban burdens of paediatric asthma incidence attributable to ambient NO _2_ pollution: estimates from global datasets. *Lancet Planet Health.* 2019;3(4):e166–78. 10.1016/S2542-5196(19)30046-4 30981709

[ref2] Anenberg SC Henze DK Tinney V : Estimates of the Global Burden of Ambient PM _2.5_, Ozone, and NO _2_ on Asthma Incidence and Emergency Room Visits. *Environ Health Perspect.* 2018;126(10):107004. 10.1289/EHP3766 30392403 PMC6371661

[ref3] Apte JS Brauer M Cohen AJ : Ambient PM _2.5_ Reduces Global and Regional Life Expectancy. *Environ Sci Technol Lett.* 2018;5(9):546–51. 10.1021/acs.estlett.8b00360

[ref4] Atkinson RW Carey IM Kent AJ : Long-term exposure to outdoor air pollution and the incidence of chronic obstructive pulmonary disease in a national English cohort. *Occup Environ Med.* 2015 Jan;72(1):42–8. 10.1136/oemed-2014-102266 25146191 PMC4283678

[ref6] Blanco-Becerra LC Miranda-Soberanis V Hernández-Cadena L : Effect of particulate matter less than 10μm (PM10) on mortality in Bogota, Colombia: a time-series analysis, 1998-2006. *Salud Publica Mex.* 2014;56(4):363–70. 10.21149/spm.v56i4.7356 25604176

[ref5] Burnett RT Pope CA 3rd Ezzati M : An integrated risk function for estimating the global burden of disease attributable to ambient fine particulate matter exposure. *Environ Health Perspect.* 2014;122(4):397–403. 10.1289/ehp.1307049 24518036 PMC3984213

[ref50] Castañeda-Orjuela C Chaparro P Solarte-Agredo I : *Primer Informe ONS. Aspectos relacionados con la frecuencia de uso de servicios, mortalidad y discapacidad en Colombia, 2011.* Bogotá (Colombia): Instituto Nacional de Salud;2015; [cited 2021 july 27];196p. Spanish. Reference Source

[ref7] Cohen AJ Brauer M Burnett R : Estimates and 25-year trends of the global burden of disease attributable to ambient air pollution: an analysis of data from the Global Burden of Diseases Study 2015. *Lancet.* 2017;389(10082):1907–18. 10.1016/S0140-6736(17)30505-6 28408086 PMC5439030

[ref9] Doiron D de Hoogh K Probst-Hensch N : Air pollution, lung function and COPD: results from the population-based UK Biobank study. *Eur Respir J.* 2019;54(1):1802140. 10.1183/13993003.02140-2018 31285306

[ref8] Dominici F Peng RD Bell ML : Fine particulate air pollution and hospital admission for cardiovascular and respiratory diseases. *JAMA.* 2006;295(10):1127–34. 10.1001/jama.295.10.1127 16522832 PMC3543154

[ref10] Efron B Tibshirani R : *An Introduction to the Bootstrap.* New York, London: Chapman and Hall:1993;456p.

[ref11] Franklin BA Brook R Arden Pope C 3rd : Air pollution and cardiovascular disease. *Curr Probl Cardiol.* 2015;40(5):207–38. 10.1016/j.cpcardiol.2015.01.003 25882781

[ref12] Gaviria C Benavides C Tangarife C : [Particulate air pollution (PM2.5 and PM10) and medical consultations due to respiratory disease in Medellín (2008-2009)] *Rev Fac Nac Salud Pública (Medellín).* 2011 [cited 2020 Dec 5];29(3):241–50. Spanish. Reference Source

[ref13] GBD 2019 Risk Factors Collaborators: Global burden of 87 risk factors in 204 countries and territories, 1990-2019: a systematic analysis for the Global Burden of Disease Study 2019. *Lancet.* 2020;396(10258):1223–49. 10.1016/S0140-6736(20)30752-2 33069327 PMC7566194

[ref14] GBD Chronic Respiratory Disease Collaborators: Prevalence and attributable health burden of chronic respiratory diseases, 1990-2017: a systematic analysis for the Global Burden of Disease Study 2017. *Lancet Respir Med.* 2020 Jun;8(6):585–96. 10.1016/S2213-2600(20)30105-3 32526187 PMC7284317

[ref15] Golub E Klytchnikova I Sánchez- Martínez G : Environmental Health Costs in Colombia. The Changes from 2002 to 2010. Washington: World Bank;2014. [cited 2020 Oct 12].49p. Reference Source

[ref16] Grisales-Romero H Nieto-López E Montealegre-Hernández N : Underlying data for ‘Local attributable burden disease to PM2.5 ambient air pollution in Medellín, Colombia, 2010–2016’. 2021. 10.5281/zenodo.4646592 PMC8564742 34745558

[ref17] Hajat A Diez Roux AV Castro-Diehl C : The Association between Long-Term Air Pollution and Urinary Catecholamines: Evidence from the Multi-Ethnic Study of Atherosclerosis. *Environ Health Perspect.* 2019;127(5):57007. 10.1289/EHP3286 31095432 PMC6791118

[ref18] Health Effects Institute: State of Global Air 2019. A special report on global exposure to air pollution and its disease burden. Boston, MA: Health Effects Institute 2019. [cited 2020 Oct 06].22p. Reference Source

[ref19] Hu LW Qian Z Bloom MS : A panel study of airborne particulate matter concentration and impaired cardiopulmonary function in young adults by two different exposure measurement. *Atmos Environ.* 2018;180:103–9. 10.1016/j.atmosenv.2018.03.001

[ref20] India State-Level Disease Burden Initiative Air Pollution Collaborators: The impact of air pollution on deaths, disease burden, and life expectancy across the states of India: The Global Burden of Disease Study 2017. *Lancet Planet Health.* 2019;3(1):e26–39. 10.1016/S2542-5196(18)30261-4 30528905 PMC6358127

[ref21] Instituto Nacional de Salud; Observatorio Nacional de Salud: Carga de Enfermedad Ambiental en Colombia. Bogotá (Colombia): Instituto Nacional de Salud;2018 [cited 2020 Jul 16].178p. Spanish. Reference Source

[ref22] Kauffman JD Adar SD Barr RG : Association between air pollution and coronary artery calcification within six metropolitan areas in the USA (the Multi-Ethnic Study of Atherosclerosis and Air Pollution): a longitudinal cohort study. *Lancet.* 2016a;388(10045):696–704. 10.1016/S0140-6736(16)00378-0 27233746 PMC5019949

[ref23] Kauffman JD Spalt EW Curl CL : Advances in Understanding Air Pollution and CVD. *Glob Heart.* 2016b;11(3):343–52. 10.1016/j.gheart.2016.07.004 27741981 PMC5082281

[ref51] Li H : Nonsymmetric Dependence Measures: The Discrete Case. 2015 [cited 2021 Jun 11]. Reference Source

[ref24] Liu S Jørgensen JT Ljungman P : Long-term exposure to low-level air pollution and incidence of chronic obstructive pulmonary disease: The ELAPSE project. *Environ Int.* 2021;146:106267. 10.1016/j.envint.2020.106267 33276316

[ref25] Murray CJ : Quantifying the burden of disease: the technical basis for disability-adjusted life years. *Bull World Health Organ.* 1994;72(3):429–45. 8062401 PMC2486718

[ref26] Nieto-López E Grisales-Romero H Montealegre NA : Effects on Morbidity and Mortality of Critical Episodes of PM2.5 in the City of Medellin, 2015. *Poster session presented at: 30st Conference of International Society of Environmental Epidemiology: Addressing Complex Local and Global Issues in Environmental Exposure and Health*; 2018 August 26-30; Ottawa, Canada. Reference Source

[ref27] Nishimura KK Galanter JM Roth LA : Early-life air pollution and asthma risk in minority children. The GALA II and SAGE II studies. *Am J Respir Crit Care Med.* 2013;188(3):309–18. 10.1164/rccm.201302-0264OC 23750510 PMC3778732

[ref28] Organización de Naciones Unidas: Manual X Técnicas Indirectas De Estimación Demográfica. New York: Naciones Unidas;1986 [cited 2019 Nov 1].318p. Spanish. Reference Source

[ref29] Piñeros-Jiménez JG Grisales-Romero H Nieto-López E : Contaminación atmosférica y sus efectos sobre la salud de los habitantes del Valle de Aburrá, 2008a-2017. Análisis de la exposición de corto y largo plazo. 2019. *Medellín (Colombia): Área Metropolitana del Valle de Aburrá* [cited 2020 Dec 1].100p. Spanish. Reference Source

[ref30] Piñeros-Jiménez JG Grisales-Romero H Nieto-López E : Contaminación atmosférica y sus efectos sobre la salud de los habitantes del Valle de Aburrá, 2008b-2015. *Medellín (Colombia): Área Metropolitana del Valle de Aburrá* 2018 [cited 2019 Dec 1].114p. Spanish. Reference Source

[ref31] Prüss-Ustün A Wolf J Corvalán C : Preventing disease through healthy environments: A global assessment of the environmental burden of disease. Geneva: World Health Organization;2016. [cited 2020 May 10].146p. Reference Source

[ref33] Ribotta B Salazar L Bertone C : Evaluations of the Life Statistics Coverage at a Subnational Level. Recent Studies in Latin America. *Rev Gerenc y Políticas Salud (Bogotá DC).* 2019 [cited 2020 Dec 5];18(36):9–12. Spanish. Reference Source

[ref32] Riojas-Rodríguez H da Silva AS Texcalac-Sangrador JL : Air pollution management and control in Latin America and the Caribbean: implications for climate change. *Rev Panam Salud Publica.* 2016;40(3):150–9. 27991972

[ref36] Rodríguez-Villamizar LA Herrera-López AB Castro-Ortiz H : Incidence of respiratory symptoms and the association with air pollution in preschoolers: a multilevel analysis. *Cad Saude Publica (Rio de Janeiro).* 2010;26(7):1411–8. Spanish. 10.1590/s0102-311x2010000700020 20694367

[ref35] Rodríguez-Villamizar LA Rojas-Roa NY Blanco-Becerra LC : Short-Term Effects of Air Pollution on Respiratory and Circulatory Morbidity in Colombia 2011-2014: A Multi-City, Time-Series Analysis. *Int J Environ Res Public Health.* 2018 Jul 30;15(8):1610. 10.3390/ijerph15081610 30061515 PMC6121387

[ref34] Romieu I Gouveia N Cifuentes LA : Multicity study of air pollution and mortality in Latin America (the ESCALA study). *Res Rep Health Eff Inst.* 2012; (171):5–86. 23311234

[ref52] Rosselli D Rueda JD : Burden of pneumococcal infection in adults in Colombia. *J Infect Public Health.* 2012 Oct;5(5):354–9. 10.1016/j.jiph.2012.04.003 23164564

[ref37] Rutstein DD Mullan RJ Frazier TM : Sentinel Health Events (occupational): a basis for physician recognition and public health surveillance. *Am J Public Health.* 1983 Sep;73(9):1054–62. 10.2105/ajph.73.9.1054 6881402 PMC1651048

[ref39] Salazar-Ceballos A Álvarez-Miño L : The effects of particulate matter 10 (PM 10) and climatological variables in hospital admissions for respiratory diseases of children in the city of Santa Marta, Colombia, 2008-2009. *DUAZARY (Santa Marta).* 2011 [cited 2020 Sep 5];8(2):127–42. Spanish. 10.21676/2389783X.210

[ref38] Salomon JA Haagsma JA Davis A : Disability weights for the Global Burden of Disease 2013 study. *Lancet Glob Health.* 2015;3(11):e712–23. 10.1016/S2214-109X(15)00069-8 26475018

[ref41] Schikowski T Adam M Marcon A : Association of ambient air pollution with the prevalence and incidence of COPD. *Eur Respir J.* 2014;44(3):614–26. 10.1183/09031936.00132213 24488569

[ref40] Schikowski T Sugiri D Ranft U : Long-term air pollution exposure and living close to busy roads are associated with COPD in women. *Respir Res.* 2005;6(1):152. 10.1186/1465-9921-6-152 16372913 PMC1352358

[ref42] Song L Smith GS Adar SD : Ambient air pollution as a mediator in the pathway linking race/ethnicity to blood pressure elevation: The multi-ethnic study of atherosclerosis (MESA). *Environ Res.* 2020 Jan;180:108776. 10.1016/j.envres.2019.108776 31639655

[ref53] Turner MC Andersen ZJ Baccarelli A : Outdoor air pollution and cancer: An overview of the current evidence and public health recommendations. *CA Cancer J Clin.* 2020;70:460–79. 10.3322/caac.21632 PMC7904962 32964460

[ref43] Villa-Garzon FA Grisales-Vargas SC Ospina-Galeano DI : Artificial Neural Networks to Mix Datasets from Particulate Matters and O3 in Medellin, Colombia. *Paper presented at: 30st Conference of International Society of Environmental Epidemiology: Addressing Complex Local and Global Issues in Environmental Exposure and Health;* 2018 August 26-30; Ottawa, Canada. Reference Source

[ref44] Wendling ZA Emerson JW de Sherbinin A : Environmental Performance Index 2020. New Haven, CT: Yale Center for Environmental Law & Policy;2020. [cited 2021 Jan 20].205p. Reference Source

[ref45] World Bank: Methodology for Valuing the Health Impacts of Air Pollution: Discussion of Challenges and Proposed Solutions. Washington DC: World Bank;2016. [cited 2020 Jan 20].59p. Reference Source

[ref47] World Health Organization: Ambient air pollution: a global assessment of exposure and burden of disease. Geneva: World Health Organization;2016 [cited 2020 Jun 14].121p. Reference Source

[ref46] World Health Organization: Methods and data sources for global burden of disease estimates 2000-2015. Geneva: Department of Health Statistics and information Systems, WHO;2017 [cited 2020 Jan 14].83p. Reference Source

[ref48] Yang Y Tang R Qiu H : Long term exposure to air pollution and mortality in an elderly cohort in Hong Kong. *Environ Int.* 2018;117:99–106. 10.1016/j.envint.2018.04.034 29730535

[ref49] Yin P Brauer M Cohen AJ : The effect of air pollution on deaths, disease burden, and life expectancy across China and its provinces, 1990-2017: an analysis for the Global Burden of Disease Study 2017. *Lancet Planet Health.* 2020;4(9):e386–98. 10.1016/S2542-5196(20)30161-3 32818429 PMC7487771

