# Supplementary material for: Local attributable burden disease to PM 2.5 ambient air pollution in Medellín, Colombia, 2010–2016
Source: F1000Res. 2021 Dec 6;10:428. Originally published 2021 May 28. [Version 2] doi: 10.12688/f1000research.52025.2 (PMC8564742; doi:10.12688/f1000research.52025.2)
Supplement: Peer review history

# Peer review: Reviewer response for version 1

**DOI:** 10.5256/f1000research.55251.r86980

**Published:** 2021-11-02

**Authors:** Carmen Ildes Rodrígues Fróes Asmus

**Affiliations:** 1 Public Health Institute, School of Medicine, Federal University of Rio de Janeiro, Rio de Janeiro, Brazil

This study aimed to determine the local burden of disease to PM _2.5_ for Medellín, one of Colombia's more populous and representative cities. It is a well-design study. The methodology is detailed step to step. The authors carried out a suitable description of the protocols and tools applied for the data quality assessment used in the study.

 This article brings important information about the impact of PM _2.5_ exposure on disease burden in a Latin American city. Also it contributes, as a feasible model, for the fulfilment of other similar studies in other Latin American cities.

Is the work clearly and accurately presented and does it cite the current literature?

Yes

If applicable, is the statistical analysis and its interpretation appropriate?

Yes

Are all the source data underlying the results available to ensure full reproducibility?

Yes

Is the study design appropriate and is the work technically sound?

Yes

Are the conclusions drawn adequately supported by the results?

Yes

Are sufficient details of methods and analysis provided to allow replication by others?

Yes

Reviewer Expertise:

Environmental health; epidemiology

I confirm that I have read this submission and believe that I have an appropriate level of expertise to confirm that it is of an acceptable scientific standard.

# Response

**Published:** 2021-11-04

**Authors:** Hugo Grisales-Romero

**Affiliations:** University of Antioquia, Colombia

Thank you very much for taking the time to review this manuscript. Your review was very valuable.

 Best regards

# Peer review: Reviewer response for version 1

**DOI:** 10.5256/f1000research.55251.r86434

**Published:** 2021-06-24

**Authors:** Pablo Enrique Chaparro Narváez

**Affiliations:** 1 Instituto Nacional de Salud, Bogotá, Colombia

The estimation of the burden of disease attributable to PM 2.5 in Medellín, in terms of DALYs, is novel in Colombia. It is constituted in scientific knowledge that contributes to the identification of the necessary measures to face this problem. It is a well-designed study.

 There are a few issues that deserve attention:

**In methods**

- To include a reference that supports the relationship between in situ neoplasms (D00-D09) and PM 2.5 (page 4)

**In results**

- In figure 1A – G some of the data described do not match: In February and April, it is not evident a higher trend towards a monthly average increase of PM 2.5 pollution during the study period (page 5); “October and November from 2010 to 2014 presented monthly averages with slight increases”, except in 2011 (page 5); For years 2015 and 2016, the trend was not downward during these months (page 5).
- The results are not presented for attributable prevalent cases to PM 2.5 pollution for in situ neoplasms (D00 – D09).
- Page 9: "with a slightly greater magnitude in the male burden than the female burden (Table 1)". This table does not discriminate by sex.
- Page 9: table 3, “female average LDBPM2.5 (UI 95%) YLLs 2,886 (82,820 - 2,952)”. Please, revise UI 95%
- Page 12: table 5, “other acute lower respiratory tract infection (J20 – J22) DALYs PM2.5 (95% UI) 1,948 (594 - 1,502)”. Please, revise UI 95%
- In figure 3 identify quadrants A, B, C, D.

I recommend to consider the following issues and address them:

- The quality of air pollutant data.
- The under-registration of RIPS. This has been presented previously in several publications (Primer Informe ONS. Aspectos relacionados con la frecuencia de uso de servicios, mortalidad y discapacidad en Colombia, 2011) (Burden of pneumococcal infection in adults in Colombia).
- The influence of garbage codes to improve the comparability of data with respect to causes of death in the estimates obtained.
- Use of the GBD 2013 methodology of the WHO and methodology GBD Institute for Health Metrics and Evaluation IHME. The GBD IHME was mentioned in strengths (page 17).

Is the work clearly and accurately presented and does it cite the current literature?

Yes

If applicable, is the statistical analysis and its interpretation appropriate?

Yes

Are all the source data underlying the results available to ensure full reproducibility?

Partly

Is the study design appropriate and is the work technically sound?

Yes

Are the conclusions drawn adequately supported by the results?

Yes

Are sufficient details of methods and analysis provided to allow replication by others?

Yes

Reviewer Expertise:

Public health, epidemiology

I confirm that I have read this submission and believe that I have an appropriate level of expertise to confirm that it is of an acceptable scientific standard.

## References

[1] : Primer Informe ONS. Aspectos relacionados con la frecuencia de uso de servicios, mortalidad y discapacidad en Colombia.2011;

[2] : Burden of pneumococcal infection in adults in Colombia. *J Infect Public Health* .2012;5(5) : 10.1016/j.jiph.2012.04.003 354-9 10.1016/j.jiph.2012.04.003 23164564

# Response

**Published:** 2021-07-21

**Authors:** Hugo Grisales-Romero

**Affiliations:** University of Antioquia, Colombia

**ANSWERS TO REVIEWER**

**COMMENT 1: **To include a reference that supports the relationship between in situ neoplasms (D00-D09) and PM 2.5 (page 4).

**ANSWER: **An exhaustive review was made of events associated with air pollution. In the review by Turner *et al.* (2020) on air pollution and cancer, a wide list of events is evidenced in addition to lung and respiratory cancer, which includes neoplasms in different organs and systems. In the appendices to the recent report from the 2019 Global Burden of Disease study published in The Lancet 2020, specific codes D00-D09 are included between specific events with specific Population Attributable Fractions.

**Reference**

 Turner, MC, Andersen, ZJ, Baccarelli, A, Diver, WR, Gapstur, SM, Pope, CA, Prada, D, Samet, J, Thurston, G, Cohen, A. Outdoor air pollution and cancer: An overview of the current evidence and public health recommendations. In: CA Cancer J Clin. 2020: 70: 460- 479. [cited 2021 Jun 25], Available from https://doi.org/10.3322/caac.21632

**COMMENT 2: **In figure 1A – G some of the data described do not match: In February and April, it is not evident a higher trend towards a monthly average increase of PM 2.5 pollution during the study period (page 5); “October and November from 2010 to 2014 presented monthly averages with slight increases”, except in 2011 (page 5); For years 2015 and 2016, the trend was not downward during these months (page 5).

**ANSWER:** We agree with the opinion of the evaluator, the corrections will be included in the second version of the manuscript, which will consist of describing the dynamics of the monthly concentrations of PM _2.5_, and it is observed that in the months of February and March of each year the highest averages were presented during the study years, except for 2012.

 Likewise, there is a greater tendency to increase the monthly average for PM _2.5_ for these months during the study period. In March 2016, the highest mean value of the entire time series of data were identified.

 On the other hand, the months of October and November from 2010 to 2016 presented monthly averages with slight increases, except for 2011 (Figure 1A-G).

**COMMENT 3:** results are not presented for attributable prevalent cases to PM 2.5 pollution for in situ neoplasms (D00 – D09).

**ANSWER: **Due to the few cases reported for codes D00-D09 in the Medellín information systems, we decided to exclude the information on prevalent cases attributable to PM _2.5_ ambient air pollution from tables 1 and 2.

**COMMENT 4: **Page 9: "with a slightly greater magnitude in the male burden than the female burden (Table 1)". This table does not discriminate by sex

**ANSWER:** The observation of evaluator is correct, the burden disease analysis by sex is presented in Table 3. The correction will be made in the second version of the manuscript.

**COMMENT 5: **Page 9: table 3, “female average LDBPM2.5 (UI 95%) YLLs 2,886 (82,820 - 2,952)”. Please, revise UI 95%

**ANSWER: **We reviewed the uncertainty interval of the female average LDBPM _2.5_ (95% UI) YLLs from table 3 and made the respective correction of upper limit of the 95% uncertainty interval. It is 2.820

**COMMENT 6: **Page 12: table 5, “other acute lower respiratory tract infection (J20 – J22) DALYs PM2.5 (95% UI) 1,948 (594 - 1,502)”. Please, revise UI 95%

**ANSWER: **We reviewed the uncertainty interval of other acute lower respiratory tract infection (J20 – J22) DALYsPM _2.5_ (95% UI) from table 5 and made the respective correction of upper limit of the 95% uncertainty interval. It is 2.594

**COMMENT 7: **In figure 3 identify quadrants A, B, C, D

**ANSWER: **Starting in the top of figure 3, from left to right, the first quadrant corresponds to C, the second quadrant to D, the third quadrant to A, and the fourth quadrant to B.

**COMMENT 8: **The quality of air pollutant data.

**ANSWER: **In previous studies carried out by the authors of this manuscript (Piñeros- Jiménez, 2018), the quality analysis of the daily concentrations of PM _2.5_ available in the Intelligent Information System and the Air Quality Surveillance Network (REDAIRE, acronym in Spanish) of the environmental authority of the metropolitan region of Aburra Valley was made. The quality of the raw data measured in each station is carried out by the Institute of Hydrology, Meteorology and Environmental Studies (REDAIRE, acronym in Spanish), who are in charge of identifying the outlier data, as well as establishing the concentration of daily averages, which are calculated with at least 75% of the hourly data valid for each day in the case of automated stations.

 For each dataset (n = 8), the percentage of days without available information was established and the missing daily concentrations of PM _2.5_ were determined using multiple imputation techniques by neural networks (Villa-Garzón, 2018). Starting from the imputed series, a representative data series of the city was assembled for each year of study, previous correlation analysis by measuring the entropy of the information based on the Bhattacharya-Hellinger-Matusita distance that allows determining the relative closeness between data sets (Li, 2015).

 The results presented in this manuscript show an overview of PM2.5 pollution in Medellín from assembled series, with the proper quality product of a necessary purification process that ideally shows the behavior of the pollutant in the city.

 Piñeros-Jiménez JG, Grisales-Romero H, Nieto-López E, Montealegre NA, Villa-Garzón FA, Agudelo R, et al. Contaminación atmosférica y sus efectos sobre la salud de los habitantes del Valle de Aburrá, 2008-2015. 2018 [Internet]. Medellín (Colombia): Área Metropolitana del Valle de Aburrá [cited 2019 Dec 1]. 114 p. Available from: https://www.metropol.gov.co/ambiental/calidad-del-aire/Biblioteca-aire/Re-analisis/ContaminacionAtmosferica_y_sus_Efectos_en_la_Salud-AMVA_2019.pdf Spanish.

 Villa-Garzon FA, Grisales-Vargas SC, Ospina-Galeano DI, Jiménez-Vargas DL, Pñieros-Jiménez JG. Artificial Neural Networks to Mix Datasets from Particulate Matters and O3 in Medellin, Colombia [Internet]. Paper presented at: 30st Conference of International Society of Environmental Epidemiology: Addressing Complex Local and Global Issues in Environmental Exposure and Health; 2018 August 26-30; Ottawa, Canada. Available from: https://ehp.niehs.nih.gov/doi/10.1289/isesisee.2018.O03.03.06

 Li H. Nonsymmetric Dependence Measures: the Discrete Case. 2015 [cited 2021 Jun 11]; Available from: https://arxiv.org/pdf/1512.07945.pdf

**COMMENT 9: **The under-registration of RIPS. This has been presented previously in several publications (Primer Informe ONS. Aspectos relacionados con la frecuencia de uso de servicios, mortalidad y discapacidad en Colombia, 2011) (Burden of pneumococcal infection in adults in Colombia).

**ANSWER: **We agree with the question raised by the evaluator in relation to the underreporting of the RIPS, which corresponds to a reality inherent to the reporting dynamics of morbidity of Colombia’s information system, as this is recognized in some previous publications. However, the advances in the timeliness of information that have been achieved during the last decade due to the efforts made by the health system at the local level, particularly Medellín has achieved less underreporting than other cities in the country.

 It should be noted that the estimation of underreporting considers the clinical history as a source of comparison for the RIPS, which as the primary document would allow comparison with the diagnoses of interest of the RIPS (capture-recapture method). However, this procedure requires access to data that is under the habeas data considerations.

 We do not consider the National Demographic and Health Survey as a source of comparison with the RIPS, due to the temporal differences between the RIPS used in the present study (2010-2016) and the information from the survey, whose most current version is that of the year 2015. Added to this is the difficulty in accessing to its anonymized database.

**COMMENT 10: **The influence of garbage codes to improve the comparability of data with respect to causes of death in the estimates obtained.

**ANSWER: **In the mortality analysis carried out in this study, the causes that have been identified as residual or garbage codes were not considered. This was limited to the groups of causes that have been identified in the literature as related to ambient air pollution and for which the parameters for calculating burden disease indicators were available, specifically the fraction of risk attributable to the population.

 To define the ICD-10 codes of the events to be included in the study, a homologation and mapping was carried out with respect to the list of causes of GBD, this activity was also validated by two clinical experts.

 Recognizing the possible information biases derived from the erroneous assignment of codes of the basic causes of death by the health personnel, the process carried out allows the comparability of the results with other studies of the environmental burden of the disease.

**COMMENT 11: **Use of the GBD 2013 methodology of the WHO and methodology GBD Institute for Health Metrics and Evaluation IHME. The GBD IHME was mentioned in strengths (page 17).

**ANSWER: **Since the first study of the global burden of disease in 1993, constant updates have been made. In 2010 they introduced significant changes in the methodology for the measurement of the base metric of the Years of Life Adjusted for Disability, which were supported by the experience of around 40 working groups in the area of ​​epidemiology, several of them providing support to the public health programs of the World Health Organization. Thus, over more than two decades this methodology has been refined and validated in national and international health analysis scenarios.

 In this study, the latest theoretical and methodological criteria defined by the WHO since 2013 were taken into account, omitting economic and social evaluations in the metric. In addition, to identify the disability weights associated with each of the events related to air pollution, the lists of the study of the global burden of diseases 1990-2017 updated by the IHME were used and the Attributable Fractions were considered. Population-based (FAP) associated with PM2.5 contamination standardized by age that were estimated for Colombia in a study carried out by the IHME (Cohen A., *et al.*, 2015).

**COMMENT 11: **Are all the source data underlying the results available to ensure full reproducibility? Partly

**ANSWER:** We do not agree with the assessment of the reproducibility of the study made by the evaluator, The following arguments support our position:

 The sources of information used were official open access sources and are identified in the text and supplementary material; ii) the critical point for the reproducibility of the study is the selection of events, and this has been detailed in the manuscript and in the references that support them, mainly the previous research carried out by this team, which evaluated the impacts of short-term exposure to atmospheric pollutants in the municipalities of the Aburrá Valley (Piñeros-Jiménez *et al.,* 2018); and iii) the manuscript describes the methodology used for the analysis of the attributable local burden disease to PM2.5 ambient air pollution, which is detailed in the study carried out by Cohen in 2015.
